# Inflammasome Proteins As Biomarkers of Multiple Sclerosis

**DOI:** 10.3389/fneur.2018.00135

**Published:** 2018-03-19

**Authors:** Robert W. Keane, W. Dalton Dietrich, Juan Pablo de Rivero Vaccari

**Affiliations:** ^1^Department of Physiology and Biophysics, Miller School of Medicine, University of Miami, Miami, FL, United States; ^2^InflamaCORE, LLC, Miami, FL, United States; ^3^Department of Neurological Surgery, The Miami Project to Cure Paralysis, Miller School of Medicine, University of Miami, Miami, FL, United States

**Keywords:** inflammasome, biomarkers, serum, caspase-1, apoptosis-associated speck-like protein containing a caspase recruitment domain, multiple sclerosis

## Abstract

Multiple sclerosis (MS) is an autoimmune disease that affects the brain and spinal cord. The inflammasome is a multiprotein complex that contributes to the innate immune response in animal models of MS as well as in patients with the disease. Important to the care of patients with MS is the need for biomarkers that can predict disease onset, disease exacerbation, as well as response to treatment. In this study, we analyzed serum samples from 32 patients with MS and 120 age-matched controls, and provide receiver operator characteristic (ROC) curves with associated confidence intervals following analyses of serum samples from patients with MS, most of which had the relapsing-remitting form of the disease, and from healthy unaffected donors, and determine the sensitivity and specificity of inflammasome proteins as biomarkers of MS. We report that caspase-1 (1.662 ± 0.6024 difference between means), apoptosis-associated speck-like protein containing a caspase recruitment domain (ASC) (407.5 ± 35.79), and interleukin (IL)-18 (78.53 + 17.86) were elevated in the serum of MS patients when compared to controls. Interestingly, the levels of IL-1β (−0.5961 ± 0.265) were lower in the MS cohort. Importantly, the area under the curve (AUC) for ASC and caspase-1 were 0.9448 and 0.848, respectively. Taken together, these data suggest that ASC and caspase-1 could be potential candidate biomarkers for MS onset.

## Introduction

Multiple sclerosis (MS) is a progressive autoimmune disorder that affects the central nervous system (CNS). Pathologically, it is characterized by demyelination in the spinal cord and brain as well as the presence of inflammatory lesions (1). The current belief is that MS is an autoimmune disease characterized by autoreactive T lymphocytes that originate in the peripheral immune system and migrate to the CNS (2). Clinically, patients with MS present blurred vision, muscle weakness, fatigue, dizziness, as well as balance, and gait problems (1). Importantly, current therapies for MS target the inflammatory response, thus highlighting the relevance of further investigation on the immune response in MS (3–5).

In the United States, alone, there are 400,000 patients with MS and about 2 million patients worldwide (1). An important area of research in the field of MS is the identification of suitable biomarkers to predict who is at risk of developing MS, biomarkers of disease progression or exacerbation, as well as biomarkers of treatment response and prognosis (1).

The inflammasome is a key mediator of the innate immune response that in the CNS was first described to mediate inflammation after spinal cord injury (6). The inflammasome is a multiprotein complex involved in the activation of caspase-1 and the processing of the pro-inflammatory cytokines, interleukin (IL)-1β and IL-18 (7). The inflammasome contributes to the inflammatory response in MS. For instance, caspase-1 and IL-1 are present in MS plaques, and these proteins are also elevated in peripheral blood mononuclear cells of MS patients (8, 9). In addition, the NOD-like receptor protein-3 (NLRP3) inflammasome has been shown to play a role in the development of experimental autoimmune encephalomyelitis (EAE) in mice. Accordingly, mice deficient in NLRP3 were protected from developing EAE, while apoptosis-associated speck-like protein containing a caspase recruitment domain (ASC) and caspase-1-deficient mice also contributed to the disease (10–12).

In this study, we provide receiver operator characteristic (ROC) curves with associated confidence intervals following analyses of serum samples from patients with MS and from healthy unaffected donors. In addition, we determine the sensitivity and specificity of inflammasome proteins to examine the potential of inflammasome signaling proteins as biomarkers of MS.

## Materials and Methods

### Participants

Subjects were enrolled in the study Prospective Collection of Samples for Research according to IRB # 201301461 approved by Schulman Associates IRB for BioreclamationIVT. In this study, we analyzed serum samples from 120 normal donors and 32 patients who were diagnosed with MS. Samples were purchased from BioreclamationIVT. The normal donor group consisted of samples obtained from 60 male and 60 female donors in the age range of 20–70 years old. These patients had no clinical diagnosis of MS or any other disease at the time of blood donation. The age range in the MS group consisted of samples obtained from patients in the age range of 24–64 years old. As shown in Table 1, 20 patients were being treated with Tysabri, 20 were diagnosed with mild to moderate relapsing remitting MS (RRMS), 2 with secondary progressive MS (SPMS) (moderate), and 10 were unspecified. Three patients were treated with Tecfidera, one with Copaxone, two with Gilenya, one with Rebif, one with Betaseron, one with Ampyra, and three untreated with a medication for MS.

**Table 1:** Subjects with multiple sclerosis (MS) gender.

Gender	Age	Diagnosis	Medications	Race	Ethnicity	Condition
Female	64	MS	Ampyra 10 mg, Losartan 50 mg, Lyrica 50 mg, Tysabri 300 mg, Amlodipine 5 mg	Caucasian		RRMS—mild

Male	50	Migraine, hypertension (HTN), MS, osteoarthritis (OA)	Baclofen 40 mg, Neurontin, Lopressor 0.25 mg, Omeprazole 40 mg, Effexor 37.5 mg, Tysabri 300 mg, Dulera 6 mg, Tizanidine 4 mg, Percocet 7.5–325 mg, Amitriptyline	Caucasian		

Female	46	MS	Tysabri 300 mg, Abilify 5 mg, Ditropan 15 mg, Effexor 150 mg, Feosol 325 mg, Ketoconazole, Klonopin 2 mg, Topamax 200 mg, Wellbutrin 300 mg, Vicodin 7.5–750 mg	Caucasian		RRMS—mild

Male	47	MS, OA, asthma	Tysabri 300 mg, Gabapentin 800 mg, Diazepam 5 mg, Vitamin D 50000 iu	Caucasian		RRMS—moderate

Female	48	MS, osteoporosis, iron anemia	Provigil 200 mg, Escitalopram 20 mg, Tysabri 300 mg, Klonopin 0.5 mg, Fiorinal-Codeine 30–50 mg	Caucasian		Secondary progressive MS (SPMS)—moderate

Female	55	MS, hypertension, depression	Effexor 200, Tysabri 300 mg, Abilify 2 mg, Cymbalta 60 mg, Modafinil 40 mg, Losartan 100 mg, Tramadol 50, Prilosec 20 mg	Caucasian		RRMS—mild

Female	47	Peanut allergy, hypercholesterolemia, hypertension, osteopenia, vitamin D deficiency, relapse remitting multiple sclerosis (RRMS), seafood allergy	Losartan 100 mg, Hydrochlorothiazide 25 mg, Amlodipine 5 mg, Epipen 0.3 mg, Atorvastatin 20 mg, Benadryl 50 mg, Ibaridronate 0.5 mg, Trivora	Caucasian		

Male	30	MS	Tecfidera 240 mg	Caucasian	Native American	RRMS—mild

Female	34	MS	Tysabri 300 mg	Caucasian		RRMS—Mild

Female	49	MS, depression	Adderall 18 mg, Amlodipine 5 mg, Detrol, Hydrochlorothiazide 25 mg, Wellbutrin 300 mg, Ocuvite, Rebif 44 mcg, Topamax 50 mg, Vitamin D3	Caucasian		RRMS—mild

Female	24	MS, hypercholesterolemia, polycystic ovary syndrome (PCOS)	Neurontin 300 mg, Percocet 5–325mg, Tizanidine 2 mg, Tysabri 300 mg, Xanax 0.5 mg, Zoloft 50 mg	Caucasian		RRMS—moderate

Male	49	MS, anxiety, depression	Clonazepam 0.5, Klonopin 0.5, Multivitamin, Neurontin 300, Wellbutrin 300, Provigil 100, Requip 0.5, Tysabri 300 mg/15 ml, Cyanocobalamin, Zoloft 100 mg	Caucasian		RRMS—moderate

Female	50	MS	Cymbalta 60 mg, Ditropan XL 10 mg, Klonopin 0.5 mg, Lexapro 20 mg, Gabapentin 500 mg, Simvastatin 10 mg, Tysabri 300 mg, Wellbutrin 150 mg	Caucasian		RRMS—Mild

Male	38	MS, eczema	Zyrtec, Advil, Tysabri	Caucasian		RRMS—mild

Female	62	MS	Tysabri, Baclofen, Ampyra, Vitamin D, Aspirin 81 mg, Mutlivitamin, Cranberry, Statin	Caucasian		RRMS—mild

Male	51	MS, hypoglycemia, asthma (unspecified)	Baclofen 10 mg, Ditropan 15 mg, Provigil 200 mg, Wellbutrin 150 mg, Multivitamin, Tysabri 300 mg	Caucasian		MS—moderate (Relapsing Remitting)

Female	38	Migraine, MS	Synthroid 375 mg, Cytomel 50 mg, Vitamins, Copaxone 40 mg	Caucasian		

Male	32	Migraine, MS	Gilenya 0.5 mg, Baclofen 20 mg, Adderall 10 mg, Klonopin 1 mg, Lyrica 75 mg, Venlafaxine 37.5 mg, Cambia 50 mg	Unknown		

Female	30	Migraine, MS	Tysabri 300 mg, Adderall 20 mg, Topamax 50 mg, Ambien 10 mg	Caucasian		

Male	36	Migraine, hypercholesterolemia, MS	Tysabri, Wellbutrin 50 mg, Lipitor 20 mg, Vitamin D3	Caucasian		

Female	48	MS, anxiety, depression, migraine	Topamax 25 mg, Keflex 250 mg, Cephalosporin 200 mg, Linzess 145mcg, Lexapro 10 mg, Flexeril 10 mg, Gilenya 0.5 mg	Caucasian		RRMS—mild

Female	55	Migraine, hypercholesterolemia, MS, asthma	Betaseron 0.3 mg, Fluoxetine 20 mg, Atorvastatin 20 mg, Multivitamin, Calcium, Vitamin D, Aspirin, Probiotic, Maxalt	Caucasian		

Female	41	Migraine, asthma, endometriosis, colitis, MS	Topamax 100 mg, Trexamet, Amitriptyline 25 mg, Clarinex, Zyrtec, Aspirin 81 mg	Caucasian	Native American	

Male	37	Migraine, MS, vertigo	Tysabri, Tyzanidine	Caucasian		

Female	41	MS	Vitamin D 2,000 U, Trazodone 50 mg, Maxalt 10 mg, Valacyclovir 1, Medrol 4 mg, Alaprazolam 0.5 mg, Lyrica 50 mg, Provigil 200 mg, Cymbalta 60 mg, Tysabri 300 mg	Caucasian		RRMS—moderate

Male	43	MS	Tysabri 300 mg	African	African-American	RRMS—mild

Female	47	Migraines, MS, psoriasis, infertility	B12, Tysabri 300mg/15ml, Xanax 0.5 mg, Advil	Caucasian		

Female	53	Migraines, MS	Tecfidera 240 mg, Ampyra 10 mg, Aranesp, Topamax 100 mg, Myrbetriq 5 mg, Linzess, Sumatriptan, Treximet 85 mg, Ritalin 10 mg	Caucasian		

Female	54	MS	Bystolic 2.5 mg, Sertraline 50 mg, Toviaz 4 mg, Tysabri 300 mg	Caucasian		RRMS—moderate

Female	47	MS	Ambien 10 mg, Clorazepate 7.5 mg, Zoloft 50 mg	Caucasian		RRMS—mild

Female	52	MS	Tecfidera 240 mg, Percocet 10–325 mg, Xanax 0.5 mg, Ambien 12.5 mg, Prevacid, Calcium 1,000 mg, Prolera, Cran Tab	Caucasian		RRMS—mild

Female	54	MS	Baclofen 10 mg, Sarella 50 mg, Ampyra 10 mg, Lipitor 40 mg, Crestor 20 mg, Vesicare 10 mg, Synthroid 0.5 mg	Caucasian		SPMS—moderate

### Simple Plex Assay

Concentration of inflammasome proteins caspase-1, ASC, IL-1β, and IL-18 in serum was analyzed as described in Ref. (13) using the Ella System (Protein System). The Simple Plex assay was analyzed by the Simple Plex Explorer software. Results shown correspond to the mean of each sample run in triplicates. The limit of quantitation for ASC was 13.1 pg/ml (lower limit of quantitation, LLOQ) and 8,000 pg/ml (upper limit of quantitation), for caspase-1, 0.66–1,000 pg/ml, for IL-1β, 0.21–840 pg/ml, and for IL-18, 0.390–3,660 pg/ml. The intra-assay mean for ASC was 47 ± 2.8 pg/ml (low QC ± SD) and 2,806 ± 152 pg/ml (high QC ± SD), for caspase-1, 5.3 ± 0.266 and 266 ± 17.8 pg/ml, for IL-1β, 8.66 ± 0.427 and 446 ± 16.5 pg/ml, and for IL-18,10.5 ± 0.524 and 569 ± 18.7 pg/ml. The inter-assay mean for ASC was 51.1 ± 5.5 and 2,551 ± 257 pg/ml, for caspase-1, 5.81 ± 0.816 and 269 ± 29 pg/ml, for IL-1β, 8.42 ± 0.342 and 410 ± 23.3 pg/ml, and for IL-18, 10.3 ± 0.882 and 587 ± 58.4 pg/ml.

### Biomarker Analyses

Prism 7 software (GraphPad) was used to analyze the data obtained from the Simple Plex Explorer Software. Comparisons between groups were carried after identifying outliers followed by determination of the area under the ROC curve, as well as the 95% confidence interval (CI). The *p*-value of significance used was <0.05. Sensitivity and specificity of each biomarker was obtained for a range of different cut-off points. Samples that yielded a protein value below the level of detection of the assay were not included in the analyses for that particular analyte.

## Results

### Caspase-1, ASC, and IL-18 Are Elevated in the Serum of MS Patients

**Figure 1:**
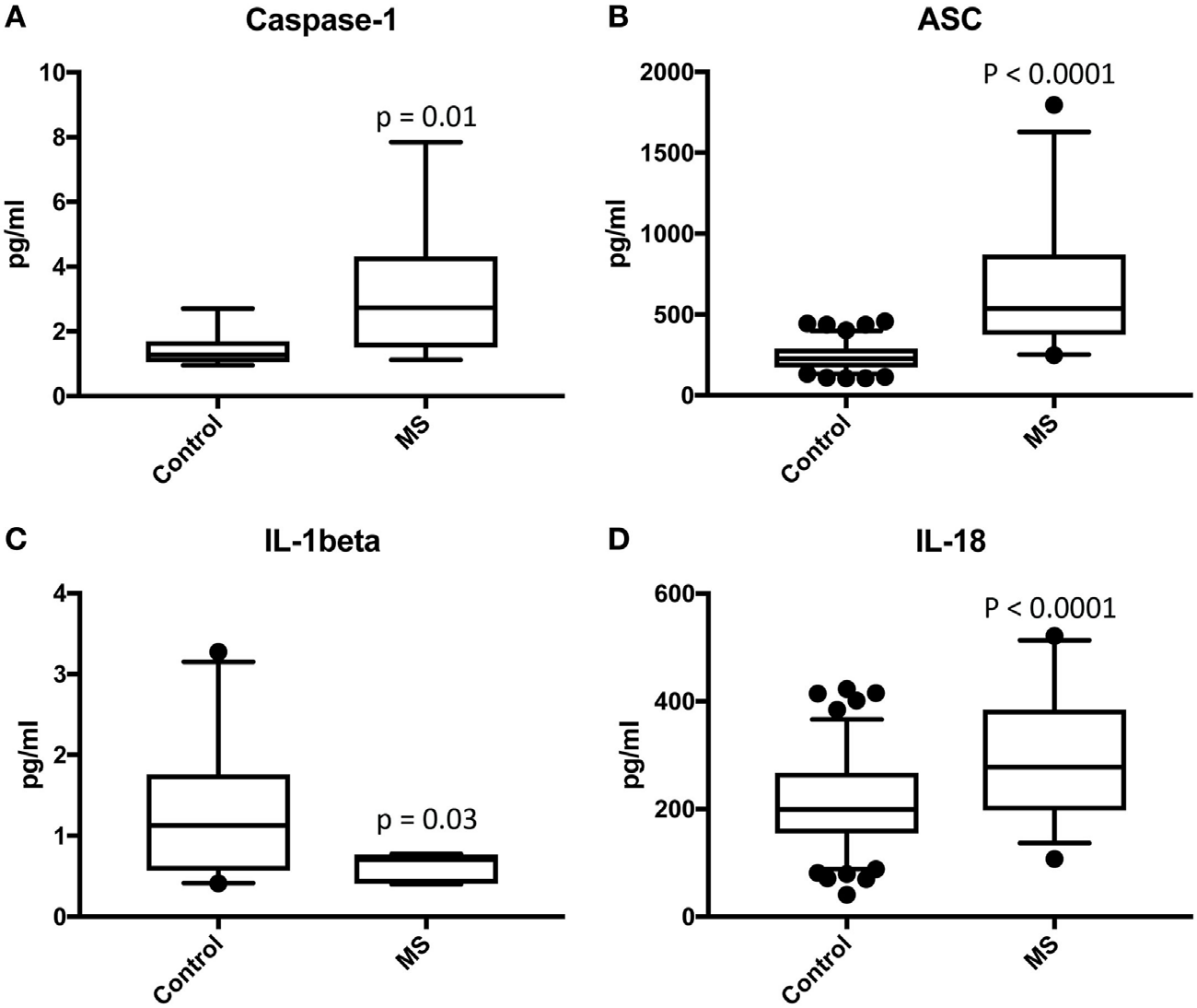
Inflammasome proteins are elevated in the serum of MS patients. Protein levels in pg/ml of caspase-1 **(A)**, apoptosis-associated speck-like protein containing a caspase recruitment domain (ASC) **(B)**, IL-1β **(C)**, and IL-18 **(D)** in serum samples from patients with MS and healthy donors. *p*-value of significance is shown above each box plot. Box and whiskers are shown for the 5th and 95th percentile. Caspase-1: *N* = 9 control and 19 MS; ASC: *N* = 115 control and 32 MS; IL-1β: *N* = 21 control and 8 MS; and IL-18: *N* = 119 control and 32 MS.

We analyzed serum samples from MS patients and compared them to serum from healthy/control individuals using a Simple Plex assay (Protein Simple) for the protein expression of the inflammasome signaling proteins caspase-1, ASC, IL-1β, and IL-18 (Figure 1). Accordingly, we found that the protein levels of caspase-1, ASC, and IL-18 in the serum of MS patients was higher than in the control group. However, the levels of IL-1β were lower in the MS than controls. These findings are consistent with previous reports indicating a role for the inflammasome in the pathology of MS (10, 11, 14).

### ASC and Caspase-1 Are Promising Serum Biomarkers of MS

**Figure 2:**
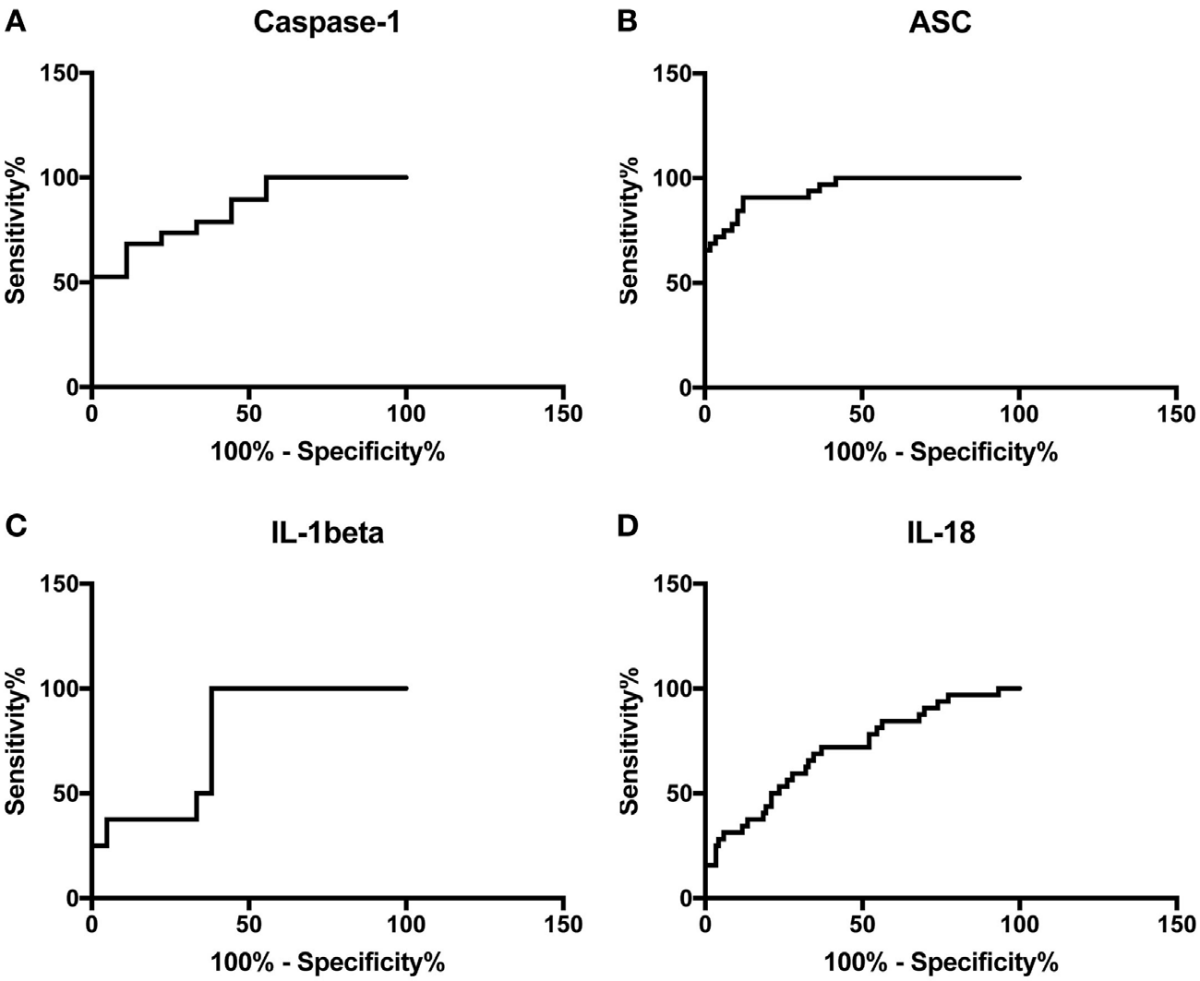
Receiver operator characteristic curves for caspase-1 **(A)**, apoptosis-associated speck-like protein containing a caspase recruitment domain **(B)**, IL-1β **(C)**, and IL-18 **(D)** from serum samples of multiple sclerosis and healthy donors.

**Figure 3:**
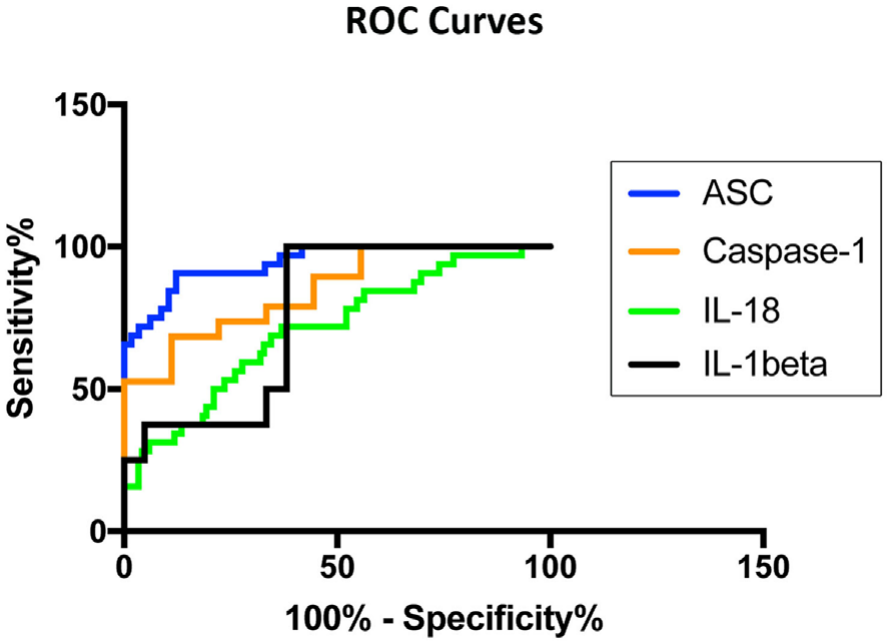
Inflammasome proteins in serum as biomarkers of multiple sclerosis (MS). Receiver operator characteristic curves for caspase-1 (orange), apoptosis-associated speck-like protein containing a caspase recruitment domain (ASC) (blue), IL-1β (black), and IL-18 (green). Caspase-1: *N* = 9 control and 19 MS; ASC: *N* = 115 control and 32 MS; IL-1β: *N* = 21 control and 8 MS; and IL-18: *N* = 119 control and 32 MS.

To then determine if these inflammasome signaling proteins have the potential to be reliable biomarkers for MS pathology, we determined the area under the curve (AUC) for caspase-1 (Figure 2A), ASC (Figure 2B), IL-1β (Figure 2C), and IL-18 (Figure 2D). Of the proteins that we measured, ASC was shown to be the best potential biomarker (Figure 3) with an AUC of 0.9448 and a CI between 0.9032 and 0.9864 (Table 2). In addition, caspase-1 with an AUC of 0.848 and a CI between 0.703 and 0.9929 is also promising biomarker of MS.

**Table 2:** Receiver operator characteristic analysis results for inflammasome signaling proteins in serum and cut-off point analyses for inflammasome signaling proteins in serum.

Biomarker	Area	SE	95% CI	*P* value	
Caspase-1	0.848	0.07394	0.703–0.9929	0.0034	
Apoptosis-associated speck-like protein containing a caspase recruitment domain (ASC)	0.9448	0.02122	0.9032–0.9864	<0.0001	
IL-1 beta	0.7619	0.0925	0.5806–0.9432	0.0318	
IL-18	0.7075	0.05216	0.6052–0.8097	0.0003	

**Biomarker**	**Cut-off point (pg/ml)**	**Sensitivity (%)**	**Specificity (%)**	**PPV (%)**	**NPV (%)**

Caspase-1	>1.302	89	56	81	71
ASC	>352.4	84	90	70	95
IL-1 beta	<0.825	100	62	50	100
IL-18	>190.1	84	44	29	91

Furthermore, the cut-off point for ASC was 352.4 pg/ml with 84% sensitivity and 90% specificity (Table 2). For caspase-1, the cut-off point was 1.302 pg/ml with 89% sensitivity and 56% specificity (Table 2). Moreover, we found that with regards to ASC for a 100% sensitivity, the cut-off point was 247.2 pg/ml with 58.26% specificity, and for 100% specificity, the cut-off point was 465.1 pg/ml with 65.63% sensitivity. In the case of caspase-1, for 100% sensitivity, the cut-off point was 1.111 pg/ml with 44.44% specificity. For 100% specificity, the cut-off point was 2.718 pg/ml with 52.63% sensitivity. Thus, these findings indicate that caspase-1 and ASC are promising biomarkers for MS.

### ASC Is a Promising Biomarker of MS Severity in Serum

**Figure 4:**
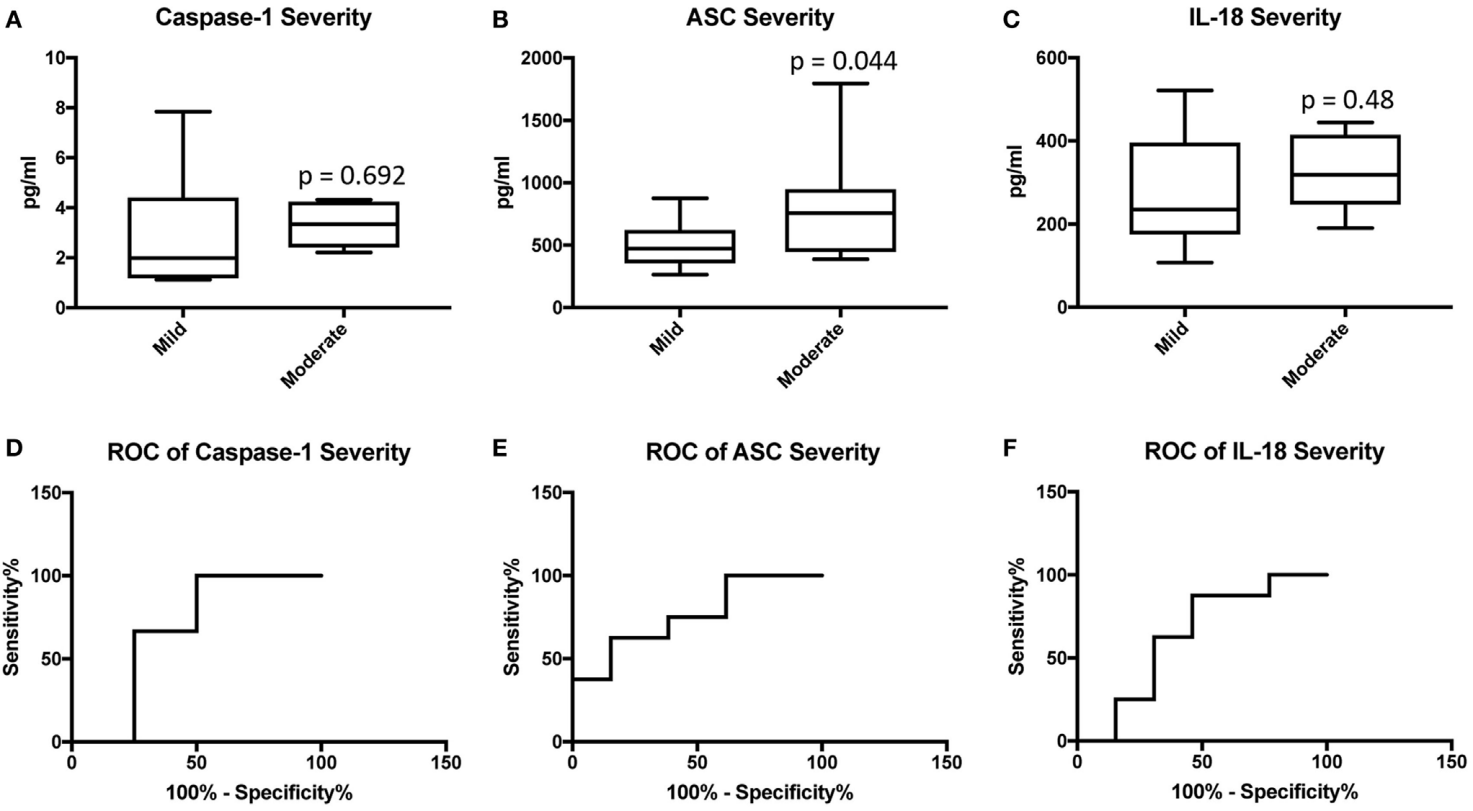
Inflammasome proteins in serum as biomarkers of multiple sclerosis (MS) severity. Protein levels in pg/ml of caspase-1 **(A)**, apoptosis-associated speck-like protein containing a caspase recruitment domain (ASC) **(B)**, and IL-18 **(C)** in serum samples from patients with MS and healthy donors. *p*-value of significance is shown above each box plot. Box and whiskers are shown for the fifth and 95^th^ percentile. Receiver operator characteristic curves for caspase-1 severity (mild vs. moderate) **(D)**, ASC **(E)**, and IL-18 **(F)**. Caspase-1: *N* = 8 mild and 6 moderate; ASC: *N* = 13 mild and 8 moderate; and IL-18: *N* = 13 mild and 8 moderate.

We then separated the groups into mild and moderate MS. Accordingly, we found that the protein level of ASC was higher in the serum of MS patients with moderate disease onset than in the mild group (*p* = 0.044) (Figure 4B), whereas the caspase-1 (Figure 4A) and IL-18 (Figure 4C) levels were not statistically different between the two groups. To then determine if inflammasome proteins can be used as diagnostic biomarkers of disease severity, we determined the AUC for caspase-1 (Figure 4D), ASC (Figure 4E), and IL-18 (Figure 4F). Of the proteins that we measured, ASC was shown to be the best biomarker with an AUC of 0.7596 and a CI between 0.5437 and 0.9756 (Table 3). Furthermore, the cut-off point for ASC was 537.5 pg/ml with 75% sensitivity and 62% specificity (Table 3). Thus, these findings indicate that ASC is a promising diagnostic biomarker for MS severity.

**Table 3:** Receiver operator characteristic analysis results for inflammasome signaling proteins in serum and cut-off point analyses for inflammasome signaling proteins in serum as markers of multiple sclerosis severity (mild vs. moderate).

Biomarker	Area	SE	95% CI	*P* value	
Caspase-1	0.6667	0.155	0.3629–0.9704	0.30	
ASC	0.7596	0.11	0.5437–0.9756	0.05	
IL-18	0.6346	0.12	0.3925–0.8767	0.31	

**Biomarker**	**Cut-off point (pg/ml)**	**Sensitivity (%)**	**Specificity (%)**	**PPV (%)**	**NPV (%)**

Caspase-1	>1.776	100	50	73	100
ASC	>537.5	75	62	76	60
IL-18	>238.2	88	54	75	73

## Discussion

Multiple sclerosis is an autoimmune disease that affects the brain and spinal cord characterized by the presence of inflammatory lesions and demyelination (1). In MS, T-cells originating in the peripheral immune response and eventually infiltrate the CNS, producing demyelination and axon degeneration (15). The source of autoimmunity in MS remains to be identified. However, anti-inflammatory therapies have been shown to be efficient in reducing disease relapse and disease progression (3–5). It is possible that in addition to infiltrating T-cells that attack the myelin, infections that target the brain may activate an inflammatory response that then goes on to attack oligodendrocytes (16). In support of the role of inflammation on MS, IL-17 has been found to play an important role in the pathology of MS. IL-17 has been found in brain lesions of patients with MS as well as in peripheral blood mononuclear cells from these patients (17, 18). As a result, studies are being conducted to target IL-17 with neutralizing antibodies (19).

Multiple sclerosis remains the most common neurological disability in young adults (20). The pathological manifestations of MS include inflammation, demyelination-neurodegeneration, axonal damage, remyelination-repair, and gliosis (20). A better understanding of the molecular mechanisms contributing to these factors represents an opportunity to develop personalized therapies. From the understanding of these molecular mechanisms, one can identify potential biomarkers that can be used to guide the diagnosis, prognosis, and treatment of MS patients.

Importantly, many publications on the field of biomarker research lack adequate measures to determine the likelihood of elevated proteins in diseased cohorts as biomarkers of a particular disease. AUC values are important in biomarker analysis for this value does not change with the prevalence of a given outcome within the cohort used for that study. Moreover, it plots the sensitivity and specificity across the range of biomarker concentrations (21). In this study, in accordance with standards of biomarker analyses, we provide AUC values with confidence intervals, sensitivities, and specificities to identify the inflammasome proteins caspase-1, ASC, IL-1β, and IL-18 as suitable biomarkers that can be used in the care of patients with MS. A perfect AUC value is 1.0, where 100% of subjects in the population will be correctly classified as having MS or not. In contrast, an AUC of 0.5 signifies that subjects are randomly classified as either positive or negative for MS, which has no clinical utility. It has been suggested that an AUC between 0.9 and 1.0 applies to an excellent biomarker; from 0.8 to 0.9, good; 0.7 to 0.8, fair; 0.6 to 0.7, poor; and 0.5 to 0.6, fail (21).

The initial manifestation of demyelination is referred to as clinically isolated syndrome (CIS). Following CIS is the clinical manifestation of MS, which is referred to as clinically definite MS (CDMS) (22). The International Advisory Committee on Clinical Trials in MS describes MS with terms, such as progressing, not progressing, active, or not active as well as RRMS, and progressive MS. Progressive MS is then subdivided into primary progressive MS (PPMS) or SPMS (23). Patients with RRMS present new or recurrent symptoms with lack of disease progression between relapses. Patients with PPMS have no prior history of RRMS and present a loss of neurological function with occasional temporary minor improvements. SPMS is used to denote patients with RRMS who are progressing (24). As a result, due to the different manifestations of the disease, there is a need to find objective biomarkers that can stratify the different clinical scenarios that MS patients present.

Pharmacogenomic biomarkers (DNA, RNA) offer great potential in the care of MS patients. Recently, several genes have been identified to be associated with the response to IFNβ treatment in MS patients, including genes encoding for type I IFN and IFN regulatory transcription factors (25, 26). In addition, other genes have been associated with a lack of response to IFNβ treatment (27). However, future studies are needed to corroborate these findings (28). As a result, to date, magnetic resonance imaging (MRI) remains the main tool used in the diagnosis and monitoring of treatment in this patient population. MRI can image the changes in the blood–brain barrier associated with inflammatory demyelination. Moreover, using the most recent criteria allows for accurate diagnosis of MS with just one scan (29). Similarly, MRI offers strong predictability on the effect of therapy on the rate of relapse (30).

The NLRP3 inflammasome plays a major role in the pathology of MS (12). Interestingly, IFN-β which is used as therapy in MS, acts as an inhibitor of NLRP3 inflammasome activation in the EAE model (31, 32). In addition, in the EAE model the pyrin inflammasome has been shown to be responsible for the production of IL-1β by hematopoietic cells following stimulation with pertussis toxin (14).

In humans, higher levels of IL-18 have been previously detected in the serum and cerebrospinal fluid of patients with MS (33, 34). In this study, we detected a statistically significant higher level of IL-18 in the serum of MS patients when compared to healthy subjects, consistent with a high AUC (0.7075).

In the EAE animal model of MS, IL-1β plays a major role in this demyelinating disease (35). However, in humans conflicting data exists regarding the levels of IL-1β in MS patients. Accordingly, some studies indicate that IL-1β is elevated in the serum and CSF of patients with MS. In this cohort, patients with RRMS were considered clinically active (36, 37); however, other studies failed to detect IL-1β in the body fluids of patients with MS (38, 39). In this study, the levels of IL-1β were significantly lower in the MS group than in the control group.

In humans, caspase-1 is present in MS plaques and is elevated in peripheral blood mononuclear cells (8, 40). In this study, we found higher protein levels of caspase-1 in the serum of MS patients consistent with a high AUC for caspase-1 (0.848). ASC is a promising therapeutic target for CNS inflammation (6, 7, 41, 42). In the EAE model of MS, ASC plays a critical role in disease exacerbation together with caspase-1 (43). In our analysis, ASC was the most promising biomarker with an AUC of 0.9448.

Thus, based on these findings caspase-1 and ASC are promising biomarker with a high AUC value and a high sensitivity. Importantly, we believe that a combination of caspase-1 and ASC as biomarkers for MS with other diagnostic criteria has the potential to increase the sensitivity of these biomarkers for MS beyond what we describe here. In addition, our findings suggest that ASC could potentially be used as a diagnostic biomarker of disease severity, since we found higher levels of ASC in the moderate MS group when compared to the mild MS group, in addition to an AUC of 0.7596.

Some clinically used biomarkers, such as serum aquaporin four antibodies (AQP4-IgG), which are used to differentiate between patients with MS and patients with neuromyelitis optica, have a median sensitivity of 62.3% with a range between 12.5 and 100%, depending on the assay used for the measurements (44).

Since the 1960s immunoglobulin (Ig) G oligoclonal bands (OCB) have been used as a classic biomarker in the diagnosis of MS (45). However, the specificity of IgG-OCB is only 61%, as a result, other diagnostic criteria is needed to clinically determine the diagnosis of MS (46), yet CSF-restricted IgG-OCB is a good predictor for conversion from CIS to CDMS, independently of MRI (47). Similar results have been obtained when analyzing IgM-OCB (48). Interestingly, IgG against measles, rubella, and varicella zoster (MRZ) are present in the CSF of MS patients, thus MRZ-specific IgG have the potential to be used as biomarkers of MS diagnosis (49). Similarly, when interpreting the results of this study, it is possible that the effects seen in these proteins is more related to an activation of the innate immune response rather than being MS-specific, especially for all other proteins, but ASC, which has a 90% specificity in this cohort of samples.

This study was carried in accordance with the standards for reporting diagnostic accuracy studies (STARD). Based on the STARD standards, the study presents certain limitations. For instance, serum used in this study was obtained from a bank of samples from MS patients. Thus, when samples were initially collected, the study of inflammasome signaling proteins was not envisioned. As a result, data collection was planned after collection of samples, and eligibility criteria did not consider the role of inflammation in this patient population. This has resulted in the diverse patient population used in this study. Therefore, a more controlled study-design is needed in the future to extend the results of the present study. In addition, most of the samples used in this study came from Caucasian patients. In future studies, we need to look at samples from other races, including Hispanics and patients of African descent. Moreover, some patients in this study were being treated for other conditions; as a result, it is possible that some of the effects on inflammasome protein expression in serum are not related to MS pathology, but to the other indications that patients presented, such as hypertension or hypercholesterolemia, among others.

It is important to highlight that samples used in this study came from patients that were treated for MS with drugs, such as Tysabri, which targets integrin receptors (50). The fact that we were able to detect higher levels of inflammasome proteins despite treatment, suggests that there is still a need for drugs to target the inflammasome and other components of the immune response as therapies for MS. Moreover, another limitation of this study is that only 3 of the 32 samples belong to patients that were not receiving any treatment for MS at the time of collection. Therefore, future studies should look at these proteins in serum samples from treatment-naïve MS patients.

In addition, future research is needed to better determine the prognostic potential of these inflammasome markers on the pathology of MS. In addition, more studies are needed looking at larger patient populations to better determine the cut-off points that will give the higher specificity and sensitivity to each biomarker as a diagnostic tool of MS disease. There is also a need to look at the different levels of inflammasome proteins in the different types of MS patients as well as in patients receiving different therapies. Importantly, in this study we have identified caspase-1 and ASC as potential biomarkers of MS pathology with high AUC values; 0.9448 and 0.848, respectively with sensitivities above 80% and in the case of ASC a specificity of 90%.

## Ethics Statement

Subjects were enrolled in the study Prospective Collection of Samples for Research according to IRB # 201301461 approved by Schulman Associates IRB for BioreclamationIVT. Samples used in this study were purchased from BioreclamationIVT.

## Author Contributions

JV contributed to the design of the experiments, he ran the experiments, performed data analyses, and wrote the manuscript. RK contributed to the design of the experiments, performed data analyses, and wrote the manuscript. WD contributed to the design of the experiments, performed data analyses, and wrote the manuscript.

## Conflict of Interest Statement

JV, RK, and WD are co-founders and managing members of InflamaCORE, LLC, and have patents on inflammasome proteins as biomarkers of injury and disease as well as on targeting inflammasome proteins for therapeutic purposes.
